# Flight performance and the factors affecting the flight behaviour of *Philaenus spumarius* the main vector of *Xylella fastidiosa* in Europe

**DOI:** 10.1038/s41598-021-96904-5

**Published:** 2021-09-02

**Authors:** Clara Lago, Elisa Garzo, Aránzazu Moreno, Laura Barrios, Antonio Martí-Campoy, Francisco Rodríguez-Ballester, Alberto Fereres

**Affiliations:** 1 Instituto de Ciencias Agrarias (ICA-CSIC), Serrano 115b, 28006 Madrid, Spain; 2 Departamento de Producción Agraria, Escuela Técnica Superior de Ingeniería Agronómica, Alimentaria y de Biosistemas (ETSIAAB), Universidad Politécnica de Madrid (UPM), Av. Puerta de Hierro, 2,4, 28040 Madrid, Spain; 3 Statistics Department, Computing Center (SGAI-CSIC), Pinar 19, 28006 Madrid, Spain; 4 Instituto de Tecnologías de la Información y Comunicaciones (ITACA), Universitat Politècnica de València (UPV), Camino de Vera, s/n, 46022 Valencia, Spain

**Keywords:** Agroecology, Entomology

## Abstract

The recent emergence of *Xylella fastidiosa* in Europe is a major threat to agriculture, including olive, almond and grape. *Philaenus spumarius* is the predominant vector of *X. fastidiosa* in Europe. Understanding vector movement is critical for developing effective control measures against bacterial spread. In this study, our goal was to set up a flight-mill protocol to assess *P. spumarius* flight potential and to analyse how different variables may affect its flight behaviour. We found that *P. spumarius* was able to fly ≈ 500 m in 30 min with a maximum single flight of 5.5 km in 5.4 h. Based on the observations, the flight potential of the females was higher in spring and autumn than in summer, and that of the males was highest in autumn. Moreover, we found that *P. spumarius* had a higher flight potential during the morning and the night than during the afternoon. Our results revealed that *P. spumarius* is likely to disperse much further than the established sizes of the infected and buffer zones designated by the EU. This knowledge on the flight potential of *P. spumarius* will be critical for improving management actions against *P. spumarius* and the spread of *X. fastidiosa* in Europe.

## Introduction

One of the major concerns in agriculture is the emergence of plant pathogens that produce severe diseases causing great losses in terms of crop production. Since we live in a globalized world, these pathogens can be transported all over the world in different ways, as is the case for *Xylella fastidiosa* Wells (1987), a vector-borne plant pathogenic bacterium native to the Americas that was introduced to the European continent through the importation of contaminated plant material^1^. The bacterium causes several plant diseases that affects many economically important crops including Pierce's Disease (PD) on grapevines, Almond Leaf Scorch Disease (ALSD) on almonds or Citrus Variegated Chlorosis (CVC) on citrus^2,3^. The bacterium is also responsible of Olive Quick Decline Syndrome (OQDS), a severe disease of olives that has been spreading northward starting from Apulia, South Italy, since 2013 at a rate of 20 km per year^4–6^. Since *X. fastidiosa* was detected in Italy, subsequent mandatory large-scale surveys throughout Europe led to its discovery in France, Germany, Spain, Portugal, and Israel. The devastating effects of the bacterium have attracted public attention in recent years, and it has become the most well-known pest, with 26% of media items^7^. Similar to many other plant pathogens, *X. fastidiosa* relies on insects as vectors to spread across the ecosystem. This bacterium is transmitted by xylem-sap feeders, and *Philaenus spumarius* L. (1758) (Hemiptera: Aphrophoridae), the meadow spittlebug, is considered the main European vector^8,9^*.*

In vector-borne pathogen systems, level of disease spread across a landscape is highly dependent on vector movement^10^. *Xylella fastidiosa* is part of a complex insect vector-bacterium-host plant pathosystem in which the understanding of vector dispersal abilities is critical for preventing the spread of the disease^11,12^. Since spittlebugs were never considered pests until the establishment of *X. fastidiosa* in Europe, knowledge of their dispersal abilities is still scarce. Adult spittlebugs are able to actively disperse, fly, crawl or leap, and there are many triggers that can provoke insect displacement, such as a defensive response, a search for resources or migration related to their life cycle^13–17^. Weaver and King^18^ observed that *P. spumarius* adults travel more than 30 m in a single flight and move as much as 100 m within 24 h from the release point. Moreover, migratory behaviour has been observed by several authors during the summer period, a migration that continues in September and differences between sexes with females migrating further and more readily than males^14,17–21^. In addition, captures of spittlebugs at heights of 15 m to 200 m above ground have been reported^18,22,23^. It is well known that many insect species use low-level jet winds to travel long distances, and these captures of spittlebugs at altitude suggest that this could be the case for spittlebugs^24–29^.

Recent mass mark recapture (MMR) field studies on spittlebugs have provided new insights into spittlebug dispersal abilities. Bodino et al.^30^, performed an MMR assay in southern Italy, sampling in concentric circles that were 10 m-120 m from the release point, and they estimated using a dispersal kernel model that 98% of the *P. spumarius* population disperses in a ratio of 400 m. Moreover, another MMR study showed that the spittlebug *Neophilaenus campestris* is able to travel more than 2 km in the field in 35 days^31^. MMR results depend on the distance of the recapture points selected, and long-distance dispersers are often not recovered; thus, there is a bias in samples collected near the origin (release point). Nevertheless, there are tools such as flight-mill devices that can overcome this problem and estimate the flight potential of insects when they are involved in long-distance migration.

Flight mills have been used since the 1950s to generate information on the flight behaviour of several orders of insects, including Hemiptera^11,29,31^. Despite the broad variety of flight-mill designs, most are based on the same principle: an insect is tethered to an arm, which is connected to some kind of stand, and then, the insect flies in a circular trajectory, allowing continuous measurement of flight parameters^32^. This tool has been applied to study the dispersal ability of insect pests with severe impacts, such as the red palm weevil^33^ and the western corn rootworm^34^. An interesting application of flight mills is their use in describing how a plant pathogen may modify the flying ability of its vector, such as the Asian citrus psyllid, when infected with *Candidatus Liberibacter asiaticus*^11^. Moreover, flight mills are commonly used to describe how abiotic factors (e.g., humidity, temperature) or biotic factors (e.g., age, sex, mated, no mated) influence insect displacement^35–38^. The vast literature on studies using flight mills shows that this tool contributes to a better understanding of insects’ flight behaviours.

There are two necessary actions that need to be implemented to contain the spread of *X. fastidiosa* according to the European Commission. These actions consist of removing all the infected and non-infected plants that belong to the same species as those of the infected ones or other susceptible species found infected in a radius of 50 m and demarcating a buffer zone 2.5 km-7 km outside the infected zone (EU 2020/1201, Articles 4 and 5, 2020). However, the long-distance dispersal of spittlebugs, which is poorly quantified to date, is a major driver of *X. fastidiosa* spread^39^. Thus, it is crucial to determine the flight potential of spittlebugs to better understand *X. fastidiosa* epidemics. Therefore, the main goals of this study were to set up a flight-mill protocol to assess *P. spumarius* flight potential and to analyse how different variables (e.g., sex, population origin, season and time of day) may affect its flight behaviour.

## Methods

### Flight mills

**Figure 1:**
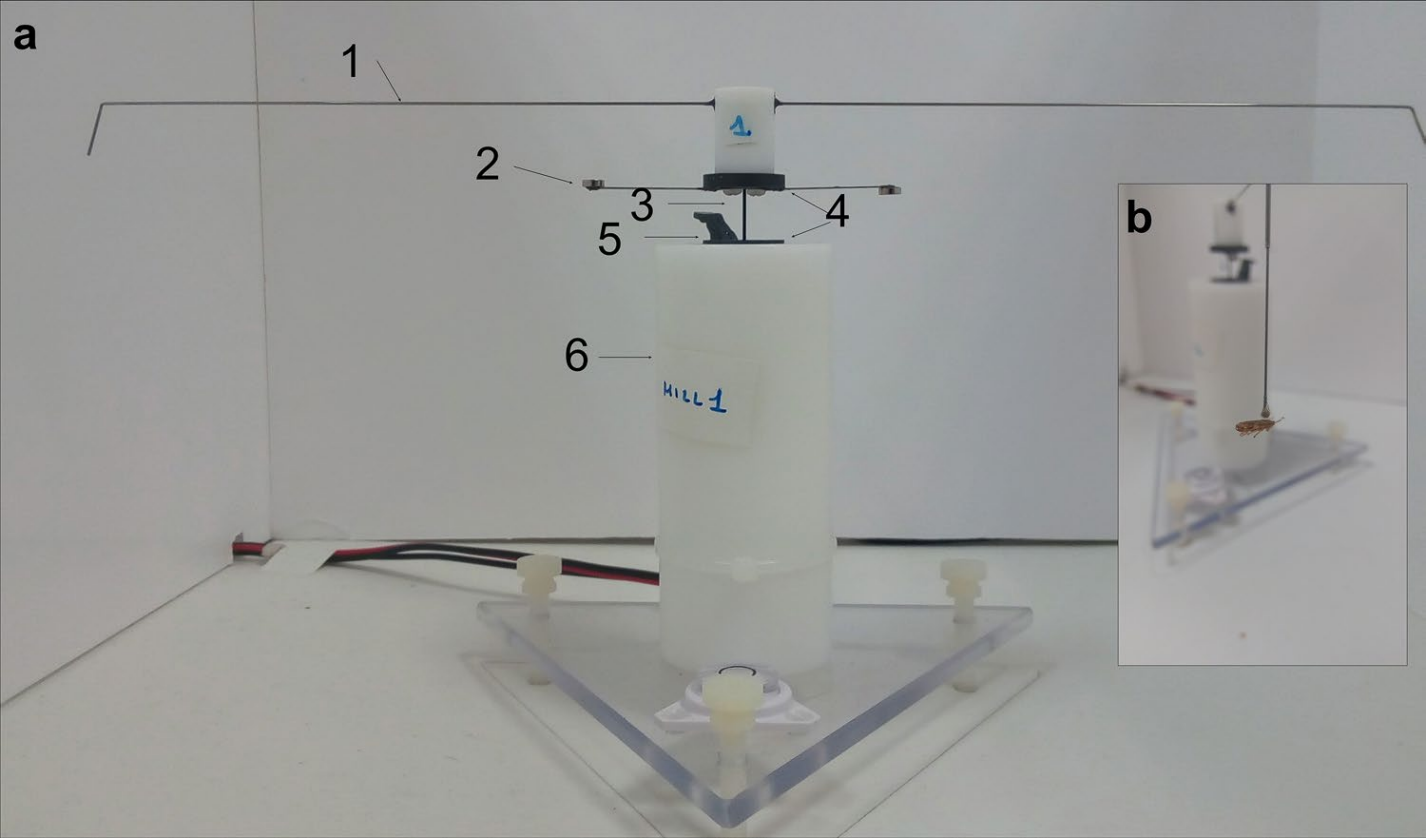
(**a**) One of the flight-mill devices used in the experiments. 1. Arm (29.6 cm) 2. The magnet detected by the sensor; the magnet on the opposite side is used to balance the arm’s weight. 3. Steel needle. 4. Magnets for levitation. 5. Hall-effect sensor. 6. Pole. (**b**) *Philaenus spumarius* glued to a pinhead and tethered to the mill’s arm.

Two commercial flight-mill devices originally designed by Jones et al.^40^, and manufactured by Crist Instruments (Insect FlyteMill, Hagerstown, MD, USA), with some adaptations to facilitate the flight of small insects, were used in the present study (Fig. 1a). Each flight mill had an arm that could be rotated. The arm was attached to the pole of the mill by a steel needle inserted in the top of the pole. The pole was constructed of Teflon to reduce the friction of the needle when spinning. Two magnets, one placed under the rotor of the arm and the other on the top of the pole, created a magnetic field that suspended the arm by means of magnetic levitation. A Hall-effect sensor was placed on the top of the pole. The sensor detected the passage of a small magnet, which was attached to the arm’s rotor; a second identical magnet was also attached on the opposite side of the arm’s rotor to balance the arm’s weight. Both ends of the arm were bent 95°, and there was a tiny hole in each end of the arm to insert a pin where the insect was attached.

A similar protocol to that described by Lago et al.^31^, was used to tether the insects to the flight mill. Insects were first anaesthetized by applying CO_2_ for 15 s. Insects were managed gently with a brush and positioned facedown in a flat surface. Then a small drop of hot melt glue (NV98591 Nivel, Leganes, Madrid, Spain) was taken with a pinhead and after three seconds the insect was glued to the pinhead by the pronotum, which is the dorsal sclerite of the prothorax of the insect (Fig. 1b). Afterwards, the insects were placed on one side of the flight mill's arm, and a suitable counterbalance of the same weight as the insect was placed on the opposite side of the arm where the insect was tethered. This allowed insects to fly in a circular trajectory. The length of the full arm (Fig. 1a) was 29.6 cm, thus the length of the circumference circumscribed by the flight mill arm was 93 cm (π*diameter = perimeter; 29.6 * 3.14 = 93; total distance per lap covered by the insect).

### Recording the flight activity of *P. spumarius*

For the recording of the flight activity of *P. spumarius,* an ad hoc acquisition system was used. This system contained a microcontroller board and two software applications for a personal computer. The microcontroller detected the moment the magnet moved over the Hall-effect sensor to record the time of the event and send it to the Mill_Recorder application. The recorded times were stored in a file as raw data that were processed later. One raw data file was created for each experiment to help manage the information. The Mill_Processor application was used to process the raw data and extract all relevant information about the flight of the insect: total number of laps, total distance travelled, total flying time, and maximum, minimum and average velocity. The flights of the insect were split into single flights^41^. The Mill_Processor software also provided details for each single flight, such as the number of laps, distance, time, and velocities, together with the average and standard deviation of these magnitudes. In addition, both software applications allowed us to set and annotate all the experimental conditions: date and time of the experiment, sex and origin of the insect, temperature (C°), atmospheric pressure (mb), relative humidity (%), light (microE), number of times an insect was attached, insect colour, and experiment duration. All the flight mill assays were conducted in a closed laboratory and controlled environmental conditions (23–25 °C).

### Initial trials

Two initial trials were performed to set up the protocol for performing the flight-mill assays and assessing the flight potential of *P. spumarius*. Insects were collected during spring 2018 as 4–5 stage nymphs in Colmenar Viejo (Madrid, central Spain) and in Sierra de Aracena (Huelva, southwest Spain). The nymphs were caged on *Sonchus oleraceus* L. plants and maintained under seminatural conditions in a greenhouse (temperature of 22.28 ± 0.23 °C with a max of 40.06 °C and a min of 7.99 °C and an RH of 54.64 ± 0.61% with a max of 99.31% and a min of 19.95%) until adulthood. *S. olearaceus* plants were collected in Morata de Tajuña (Madrid, Spain) on April 3rd, 2018 with permission of the Plant Health Department of the Spanish Ministry of Agriculture. Tests were conducted under artificial fluorescent light (10 μE m^−2^ s^−1^). Flight-mill devices were completely covered by white polyspan boxes to avoid visual stimulation disturbance and air currents. The boxes were opened on the top for ventilation and to allow illumination. Atmospheric pressure was also recorded (mean ± SE: 941 ± 0.50 hPa with a max of 950 hPa and a min of 926 hPa).

We observed that *P. spumarius* does not fly when losing tarsal contact with the ground. For insects without a tarsal reflex not all individuals start to fly when tethered to flight-mill devices and they tend to start and stop flying freely several times during the recording period^33,42^. Thus, the aim of the first trial was to establish the time lapse from when an insect was tethered to the mill to when it started flying. In this first trial, we also established the criteria to classify insects as flyers or non-flyers and the time when the recordings should be finished. The second trial was to establish the maximum length of a complete spin (or lap). For the first trial, insects were attached to the mill, and the number of seconds until they started to fly was recorded. Individuals who did not fly after an hour were directly discarded. Then, the percentage of individuals that started to fly after each 5 min interval was recorded. To establish the maximum time taken to complete a lap when flying, the duration of all the laps from 20 different, randomly selected recordings was recorded. Then, the laps were split depending on their duration in intervals of 10 s. Laps longer than 60 s were directly discarded in our initial trials because it was observed by naked eye that when an insect took more than 60 s to complete one lap, the individual always stopped flying. We also observed that when the software recorded laps shorter than 1 s, the insect had stopped flying above the magnetic sensor; thus, laps of less than 1 s were directly discarded.

With all the information obtained in the initial trials, we established the final protocol to perform the final set of flight-mill assays and selected the following flight parameters to estimate the flight performance of *P. spumarius*: (1) Flight incidence: the ability of a given insect to perform a successful flight (Yes/No). According to the preliminary trials, we discarded insects that did not fly within the first 15 min. (2) Number of single flights: in the same recording, insects could perform several flights, and a newly started flight was considered in the recording when more than 20 s elapsed between consecutive sensor readings in one lap. When an insect spent more than 15 min to complete one lap, we considered the recording over. (3) Distance travelled: sum of the distance covered by all flights. (4) Flight duration: sum of the duration of all flights. (5) Mean speed: mean speed of all the individual flights.

Once the protocol was established, two further experiments were conducted to study the seasonal and daily flight-activity patterns of *P. spumarius*.

### Seasonal patterns in the flight activity of *P. spumarius*

These assays were carried out from March 29th to November 20th in 2019 in the laboratory under artificial fluorescent light (10 μE m^−2^ s^−1^), and atmospheric pressure was also recorded (mean ± SE: 939.4 ± 0.21 hPa with a max of 949.0 hPa and a min of 924.0 hPa). Trials were performed at different times throughout each day between 9:00 and 17:00. Based on preliminary results and to maximize the sample size, insects were replaced after 15 min of no-flight activity. *Philaenus spumarius* nymphs were collected during spring 2019 in Colmenar Viejo and Sierra de Aracena. These two regions were selected because of their noticeable differences in climate. For example, in Aracena, the mean annual temperature in 2019 was much higher than that in Colmenar (18.2 °C and 13.8 °C, respectively); thus, *P. spumarius* nymphs started to emerge in early March in Aracena, while emergence in Colmenar was delayed until mid-April. Because of this difference in phenology, we considered population origin as a factor in the analysis. Insects were maintained as explained in the initial trial section. In this assay, we studied the effect of sex, population origin and season of the year on the five flight parameters previously described (flight incidence, number of flights, distance travelled, flight duration, and mean speed). The seasons were divided into three categories: (1) spring: 29th of March- 20th of June; (2) summer: 21st of June- 3rd of September; and (3) autumn: 4th of September-20th of November. The three seasons selected matched the different population peaks of the adults present in olive orchards in the mid-southern part of the Iberian Peninsula (Spain)^43^.

### Daily patterns in the flight activity of *P. spumarius*

These assays were carried out from October 2019 to March 2020 in the laboratory under the controlled conditions as explained before. The atmospheric pressure was also recorded (Mean ± SE: 942 ± 0.50 hPa with a max of 958.0 hPa and a min of 940.0 hPa). The *P. spumarius* insects were obtained from a continuous indoor rearing colony following the protocol described by Morente et al.^44^. All the individuals tested were from Colmenar Viejo and had similar ages. Individuals were caged on *S. oleraceus* and maintained in a walk-in growth chamber with a temperature of 24:20 °C D/N, a humidity of ca. 70% and a photoperiod of 14:10 L/D. The effect of sex and daily pattern (morning, afternoon and night) on the flight activity of *P. spumarius* was studied. Several trials were performed at different times throughout the day. The flight-mill tests were divided into three different times of day: (1) morning 7:00–14:00, (2) afternoon 14:00–20:00, and (3) night 20:00–7:00. The flight-mill tests conducted during the morning and afternoon were performed under artificial LED light (30 × 1.4 cm, 6000 K, 6 W 600 lumens and 125 µm/Mol^−1^) placed on top of the polyspan boxes to simulate natural light conditions. To maintain dark conditions during the night tests, the flight-mill boxes were covered with a polyspan cover, and the light was switched off. The insects’ cages were covered with a black blanket until the tests started. To attach the insects to the mills, they were collected with a mouth vacuum and introduced into an Eppendorf tube covered by black tape. Then, CO_2_ was applied to an individual for 20 s to ensure it remained completely anaesthetized during the tethering attachment process. The attachment process was conducted under infrared LED light following the protocol explained above (BRI 125 IR Red 250 W 230–250 V). Once the insects were tethered to the flight mill, the light was switched off. In this assay, we studied the effect of time of day and sex on the different flight parameters (flight incidence, number of flights, distance travelled, flight duration and mean speed).

### Statistical analysis

Flight incidence, number of flights, distance travelled (m), flight duration (s) and mean speed (m/s) were selected as the dependent variables to estimate the flight performance of *P. spumariu*s in both assays. In the seasonal pattern assay, we studied the effect of sex (male and female), origin (Madrid and Huelva) and season (spring, summer and autumn) on these parameters. The flight incidence was studied through a generalized linear model (GLMz) with a binomial distribution of errors and link logit. The number of flights, distance travelled, flight duration and mean speed (m/s) were analysed through a general linear model (GLM) after the transformation of the data (log_10_ (x + 1)) to fit a Gaussian distribution. In the daily pattern assay, the effect of sex and time of the day (morning, afternoon and night) was studied on the same flight parameters using the GLMz and GLM. The models were analysed by maximum likelihood, and the most parsimonious model was selected relying on the R-squared value for the GLM and on AIC (Akaike Information Criterion) for the GLMz^45,46^. ANOVA tests with post hoc Tukey’s HSD tests were performed to study the differences between the groups. Finally, a paired correlation test (Pearson) between the flight parameters was performed. All analyses were performed with IBM SPSS Statistics for Macintosh, Version 25.0 software.

### Statement on experimental research involving plants

Our study on experimental research involving plants complies with all the relevant institutional, local, national, and international guidelines and legislation. The use of *Sonchus oleraceus* L plants on the present manuscript were collected in Morata de Tajuña (Madrid, Spain) on April 3rd, 2018 with permission of the Plant Health Department of the Spanish Ministry of Agriculture. Voucher specimens of *S. oleraceus* plants described in the manuscript were deposited in the public herbarium at ICA, CSIC, Madrid and were correctly identified by the botanist Jose Manuel Martin (Department of Plant Protection, ICA, CSIC, https://www.ica.csic.es/index.php/en/research/plant-protection-department).

## Results

### Initial trials

The aims of these initial trials were to set up the time intervals in which most of the *P. spumarius* individuals started to fly and to establish the end of a recording and the minimum time threshold for completing a full spin or lap. A total of 25 out of 40 (62.5%) individuals tested began a flight in less than one hour after attachment to the flight mill. We found that 80% of these 25 individuals started to fly within the first 15 min after being attached to the flight-mill arm. Thus, we considered for further studies individuals that did not fly within the first 15 min to be non-flyers. Moreover, for flying individuals, we considered that the recording was over after a 15 min pause.

From the initial trials, a total of 4103 laps from 20 different recordings were split in 10 s intervals depending on their duration. Most of the laps (4005 out of 4103; 97.61% of the total laps) lasted between 1 and 10 s. A total of 71 laps (1.73%) lasted for 11–20 s; 14 (0.34%) lasted for 21–30 s; 6 (0.15%) lasted for 31–40 s; 3 (0.07%) lasted for 41–50 s and only 4 (0.1%) lasted for 51–60 s. Accordingly, we found that most of the insects were able to complete a full spin (or lap) within 10 s. Another observation was that laps lasting between 10 and 20 s occurred when the insect was about to stop flying. Additionally, when the system recorded laps lasting longer than 20 s the insect stopped flying and the flight was considered finished. Thus, when an insect spent more than 20 s completing one lap, the software recorded the end of a flight.

### Seasonal patterns in the flight activity of *P. spumarius*

**Figure 2:**
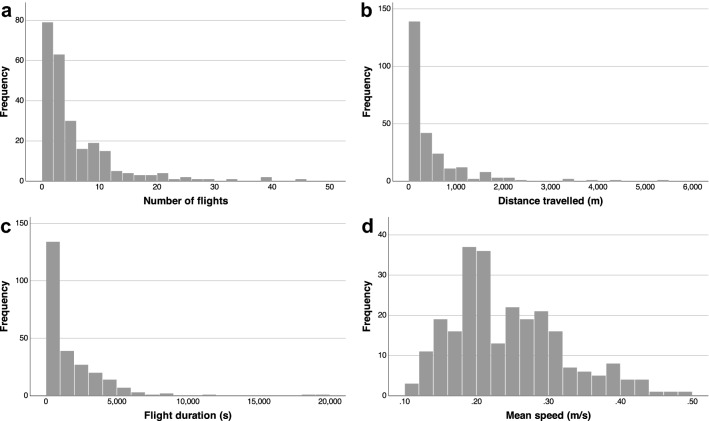
Frequency distribution of all the flight parameters studied in the assay in relation to the seasonal patterns in the flight activity of *P. spumarius*. Number of individuals studied: 250. (**a**) Number of flights performed. (**b**) Distance travelled (m). (**c**) Flight duration (s). (**d**) Mean speed (m/s).

This study investigated the influence of season, population origin and sex on the flight activity of *P. spumarius*. From a total of 432 individuals tested, 250 of them performed successful flights (57.87%). As shown in Table 1, the mean distance travelled was 459 m with a maximum of 5.5 km in a 5.4 h single flight. The remaining descriptive statistics of the flight parameters studied are shown in Table 1. The distributions of the flight parameters are shown in Fig. 2.

**Table 1:** Mean ± SE, median and maximum values of the number of total flights, distance travelled, flight duration and mean speed in relation to the influence of season, sex, and population origin on the flight activity of *P. spumarius*.

	Mean ± SE	Median	Maximum
Number of flights	5.52 ± 0.43	3	44
Distance travelled (m)	459.00 ± 45.39	192.03	5468
Flight duration (s)	1712.59 ± 154.63 (≈29 min)	902.06 ≈ 15 min	19,547 (≈5.4 h)
Mean speed (m/s)	0.24 ± 0.004	0.22	0.49

The season and the sex significantly impacted the following flight parameters studied: flight incidence (best-fitted model (binomial): sex, season and the interaction between them, chi-squared = 13.61, df = 5 and *p*-value = 0.018); distance travelled (best-fitted model (Gaussian): interaction between sex and season, R^2^-adjusted = 0.06, F = 3.99, df = 5 and *p*-value = 0.002); flight duration (best-fitted model (Gaussian): interaction between sex and season, R^2^-adjusted = 0.04, F = 2.91, df = 5 and *p*-value = 0.014); and mean speed (best-fitted model (Gaussian): sex, season and the interaction between them, R^2^-adjusted = 0.06, F = 4.17, df = 5 and *p*-value = 0.001). The origin of the insects’ populations did not significantly impact any of the parameters studied. Neither the season, the sex nor the population origin significantly impacted the number of flights performed; thus, this flight parameter was not considered in describing the flight performance of *P. spumarius.*

**Figure 3:**
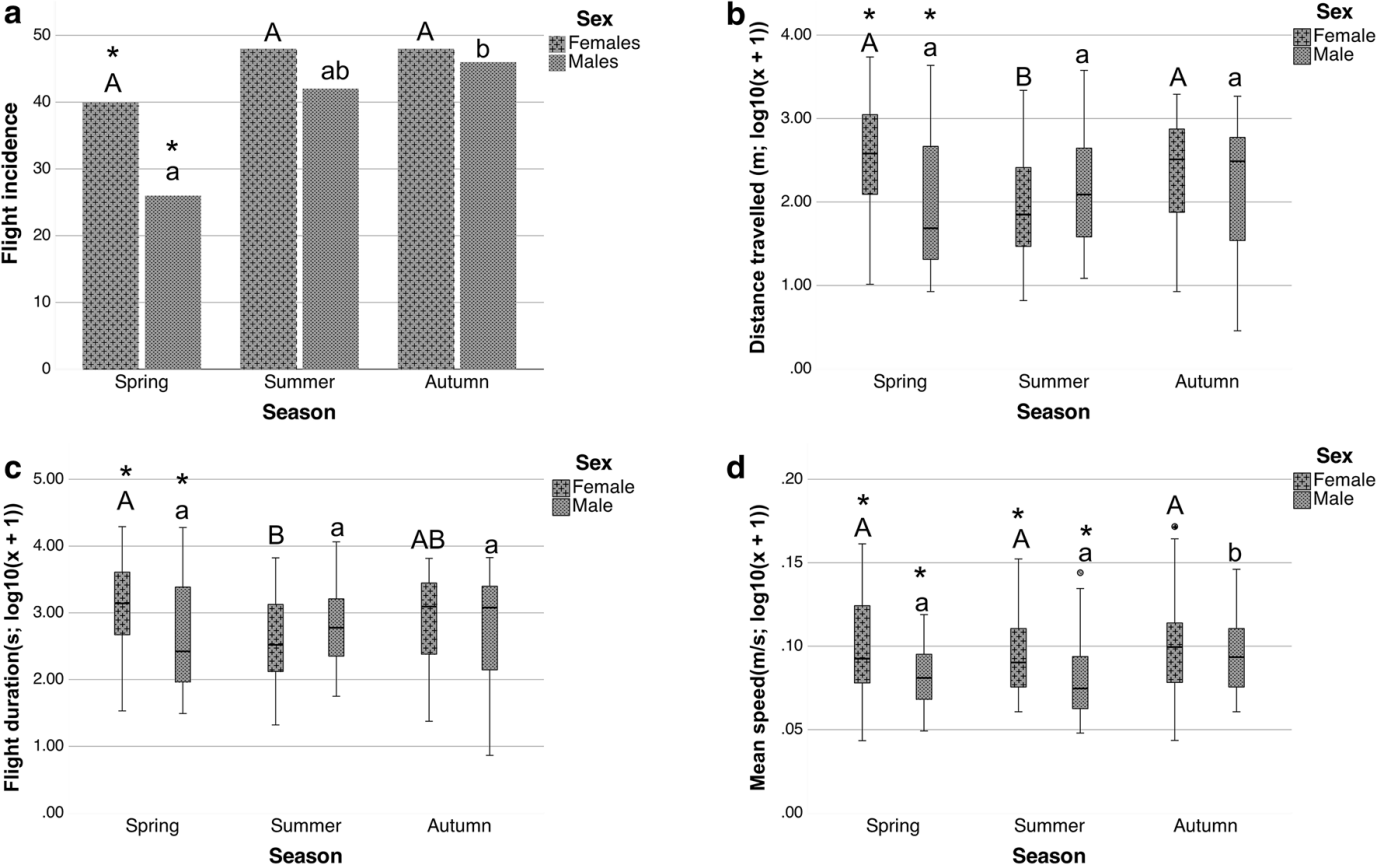
Seasonal pattern in flight activity of *P. spumarius*: Comparisons between sexes (males and females) and between seasons (spring, summer and autumn) for the flight parameters studied. (**a**) Comparisons for flight incidence. (**b**) Comparisons for distance travelled (m). (**c**) Comparisons for flight duration (s). (**d**) Comparisons for mean speed (m/s). Distance travelled, flight duration and mean speed were transformed by (log_10_(x + 1)). The capital letters refer to significant differences between the three seasons for females, and the lowercase letters refer to significant differences for males. Groups accompanied by the same letter are not significantly different. * indicates significant differences between males and females in each season. In charts b, c and d, the horizontal black lines denote median values, boxes extend from the 25th to the 75th percentile of each group’s distribution of values, and vertical extending lines denote the range of values.

When comparing the differences between seasons in terms of the flight parameters of the males, it was observed that the flight incidence was significantly lower in spring than in autumn (*p*-value = 0.018) with no significant differences with that in summer (Fig. 3a). The mean speed was significantly higher during autumn than during spring (*p*-value = 0.03) and summer (*p*-value = 0.01) (Fig. 3d). These results show that the flight performance in the males was higher in autumn than in the other seasons. For the females, the distance travelled was significantly lower during summer than during spring (*p*-value < 0.001) and autumn (*p*-value = 0.034) (Fig. 3b). Similarly, the flight duration was significantly lower in summer than in spring (*p*-value = 0.001), with no significant differences with that in autumn (Fig. 3c). Thus, the flight performance of the females was lower in summer than in the other seasons.

Moreover, when we compared sexes during the three different seasons, it was observed that during spring, the flight performance was significantly lower for males than for females (Fig. 3a–d) flight incidence (*p*-value = 0.035), distance travelled (*p*-value = 0.004), flight duration (*p*-value = 0.015) and mean speed (*p*-value = 0.008). In summer, the mean speed was significantly lower for males than for females (*p*-value = 0.011), there were no significant differences between sexes for the remaining flight parameters (Fig. 3a–d). In autumn, there were no significant differences between the sexes for any of the flight parameters studied (Fig. 3a–d). Thus, the flight performance was lower for males than for females during spring; however, the flight performance increased during the season until it was similar for both sexes in autumn.

**Figure 4:**
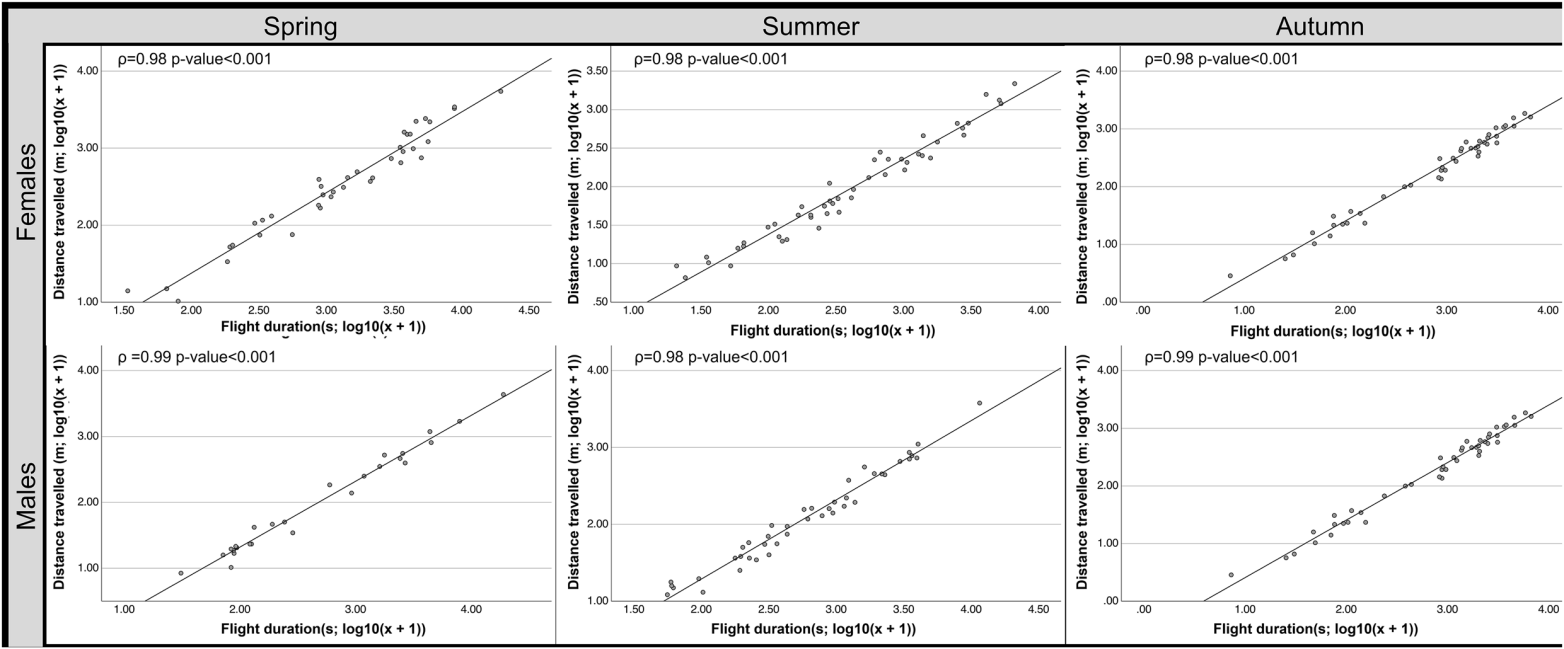
Seasonal patterns in flight activity of *P. spumarius*: Dispersal charts from correlation tests between the distance travelled and the flight duration for males and females during the three seasons (spring, summer, autumn). Both flight parameters were transformed by (log_10_(x + 1)). ρ values and *p*-values are also shown.

The correlation tests showed that the distance travelled and the flight duration were highly and positively correlated during the three seasons and in both sexes (Fig. 4); thus, the longer the flight duration was, the longer the distance travelled. For the females, there was a negative correlation between the mean speed and the number of flights during the three seasons (spring: ρ = − 0.417 *p*-value = 0.007; summer: ρ = − 0.289 *p*-value = 0.046; autumn ρ = − 0.531 *p*-value < 0.001). For the males, these two parameters were not correlated for any of the seasons. The correlation tests showed that neither the number of flights nor the mean speed were significantly correlated with the flight duration or the distance travelled.

### Daily patterns in the flight activity of *P. spumarius*

This study focused on the influence of the time of day and sex on the flight activity of *P. spumarius*. A total of 92 individuals were tested, and 60 of them performed successful flights (65.22%). As shown in Table 2, the mean distance travelled was 531 m, similar to that observed in the previous assay on seasonal patterns of flight activity (459 m, Table 1). Furthermore, the maximum distance travelled recorded was 2.2 km in 2.6 h. The remaining descriptive statistics of the flight parameters studied are shown in Table 2. Distributions of the flight parameters studied were similar to those in the previous assay.

**Table 2:** Flight parameter descriptors for the insects used in the study in terms of the influence of moment of the day and sex on *P. spumarius* flight potential.

	Mean ± SE	Median	Maximum
Number of flights	11.50 ± 1.69	6.00	61
Distance travelled (m)	530.98 ± 67.60	317.00	2174 (≈ 2.2 km)
Flight duration (s)	2086.40 ± 280.70 (≈ 35 min)	1390.00 ≈ 23.2 min	9367 (≈ 2.6 h)
Mean speed (m/s)	0.23 ± 0.009	0.21	0.41

Mean ± SE, median and maximum values of the number of total flights performed, distance travelled, flight duration and mean speed are shown.

The time of day and the sex had a significant impact: distance travelled (best-fitted model (Gaussian): sex and season with no interactions, R^2^-adjusted = 0.24, F = 7.15, df = 3 and *p*-value < 0.001) and flight duration (best-fitted model (Gaussian): sex and season with no interactions, R^2^-adjusted = 0.23, F = 6.77, df = 3 and *p*-value = 0.001).

There were differences between time of day when the data for both sexes were pooled. The distance travelled was significantly higher during the morning (*p*-value = 0.036) and during the night (*p*-value = 0.002) than during the afternoon with no significant differences between the morning and the night. Similarly, the flight duration was significantly lower during the afternoon than during the morning (*p*-value = 0.036) and the night (*p*-value = 0.004) with no significant differences between morning and night. Therefore, *P. spumarius* adults were able to perform flights during the night, showing higher flight performance during the night and the morning than during the afternoon.

Moreover, there were differences between the males and females when the data of the three times of day were pooled: the distance travelled (*p*-value = 0.017) and the flight duration (*p*-value = 0.013) were significantly higher for females than for males. Thus, the flight performance was higher for females than for males.

**Figure 5:**
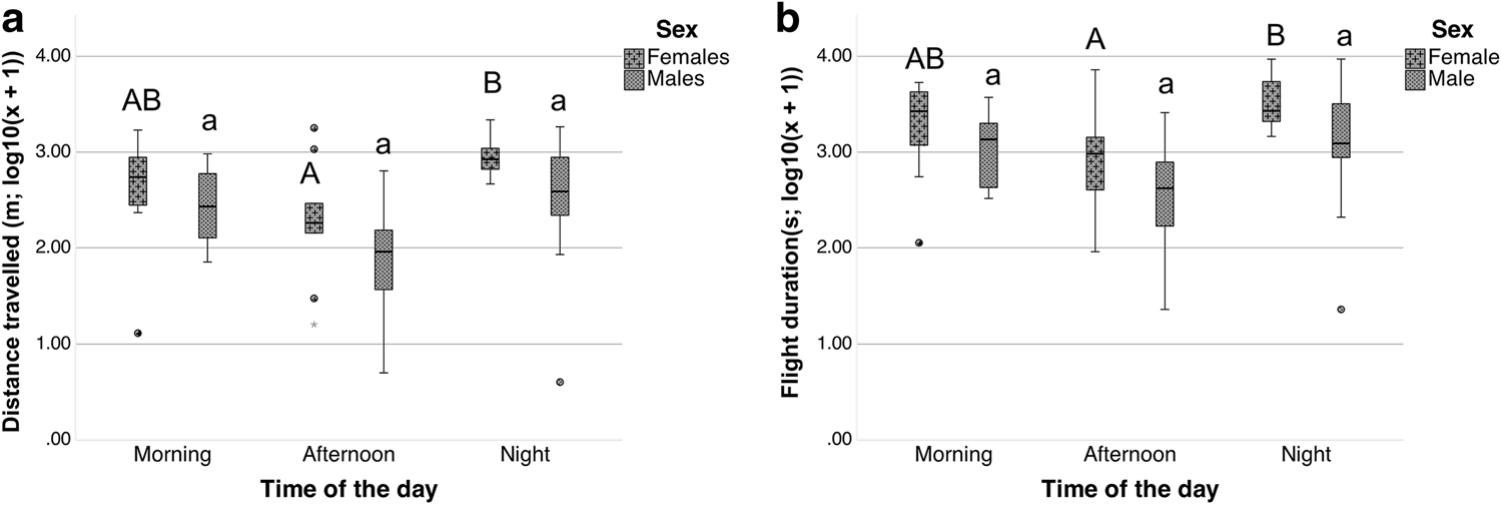
Daily patterns in flight activity of *P. spumarius*: Comparisons between sexes (males and females) and between the different times of day (morning, afternoon and night) for the flight parameters studied. (**a**) Comparisons for distance travelled (m). (**b**) Comparisons for flight duration (s). Both parameters were transformed by (log_10_(x + 1)). The capital letters refer to significant differences between the three times of the day for the females, and the lowercase letters refer to significant differences for the males. Groups accompanied by the same letter are not significantly different. The horizontal black lines denote median values, boxes extend from the 25th to the 75th percentile of each group’s distribution of values, and vertical extending lines denote the range of values.

There were differences in the flight performance of *P. spumarius* during the day depending on sex. For the males, there were no significant differences between the three times of day for any of the flight parameters studied (Fig. 5a,b). However, for the females, the distance travelled (*p*-value = 0.016) and the flight duration (*p*-value = 0.018) were significantly higher during the night than during the afternoon with no significant differences with during the morning (Fig. 5a,b). Therefore, while the flight performance of the females changed throughout the day, that of the males was similar throughout the day.

**Figure 6:**
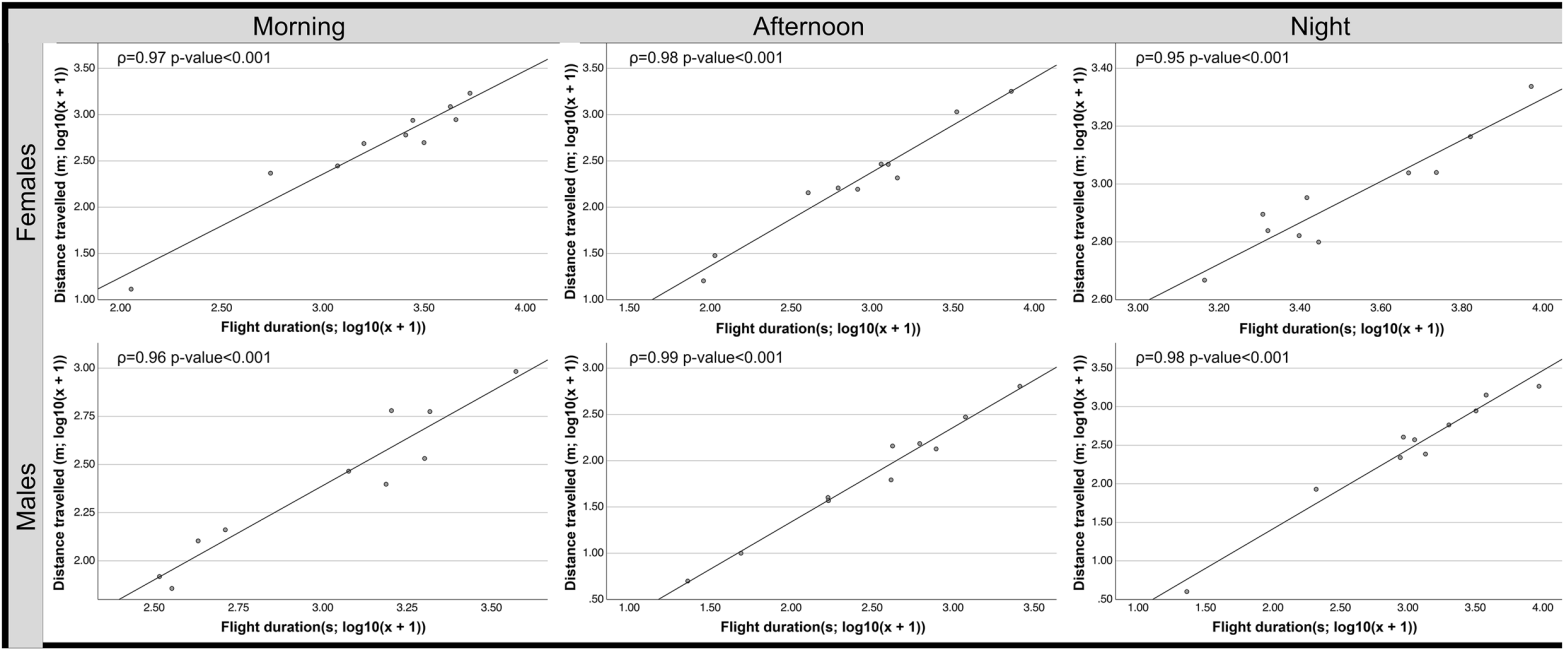
Daily patterns in flight activity of *P. spumarius*: Dispersal charts from the correlation tests between the distance travelled and the flight duration for the males and females during the three times of day (morning, afternoon and night). Both flight parameters were transformed by (log_10_(x + 1)). ρ values and *p*-values are also shown.

Similarly, as observed in the previous assay, the correlation tests showed that the distance travelled and the flight duration were positively correlated during the three times day for both sexes (Fig. 6) thus, the longer the flight duration was, the longer the distance travelled. The correlation tests showed that neither the number of flights nor the mean speed were significantly correlated, and these two factors were not correlated with the flight duration or the distance travelled.

## Discussion

*Philaenus spumarius* is the most widespread xylem-sap feeder insect in Europe and could significantly contribute to the long-distance dispersal of *X. fastidiosa*^18,39,47–49^. In fact, a recent modelling study by Strona et al. ^50^, shows that even very low probabilities of long-distance dispersal of infectious vectors dramatically increase the possibility of disease outbreaks caused by *X. fastidiosa* in olive groves in Andalusia (southern Spain) with a much larger effect compared to the pathogen’s intrinsic infectivity.

Migratory journeys and dispersal abilities of insect vectors have profound implications for the spread of vector-borne diseases^10,51–53^. Our findings provide evidence that adults of *P. spumarius* have great flight performance even in the absence of wind since the mean flight duration was almost 500 m in half an hour of flight (seasonal flights: 459 m in 29 min; daily flights: 531 m in 23 min) with a maximum distance travelled of 5.5 km in a single 5.4 h continuous flight. It is important to remark that based on preliminary results individuals that didn’t start flying after 15 min where discarded and that the end of a recording was established after a 15 min pause. This means that the total flight durations and the distances in the study are tied to this rule and these parameters could reach higher values in the field. Furthermore, some of the individuals could fly after a 15 min pause, thus distance travelled could be longer in the field. In addition, the distance travelled could be even further for migratory flights up in the atmosphere where low-level jet winds will increase speed and net distance per minute of flight. In both the seasonal and daily flight assays, the flight duration was highly and positively correlated with the distance travelled, but none of these parameters were correlated with the mean speed, suggesting that *P. spumarius* individuals are able to perform long continuous flights and travel long distances while maintaining a constant speed regardless of flight duration. In fact, as illustrated in Fig. 2b the frequency distribution of the distance travelled has a heavy-tailed distribution (characterized by Lèvy walks), suggesting the existence of some long distance flyers in the population as described for aphids^54^. Moreover, all the flight parameters studied were highly variable among all the insects studied (Fig. 2 and Tables 1, 2). As observed for many other insect species, flight performance heterogeneity in a population does not seem to be rare and suggests that some individuals are inclined towards dispersive flight, contributing to population expansion, while others are less mobile^55–57^. Long distance flights are energetically demanding and impose costs in terms of lower survival and/or reduced lifetime or reproductive success^52^. However, the primary driver of the evolution of long‐range insect migration is typically assumed to be escape from environmental conditions incompatible with development^52^. Long‐range insect migration will always represent a bet with potential rewards or penalties^58^. Thus, further experiments to estimate the costs of dispersal are needed to understand the risk posed by individuals that disperse long distances.

Several authors have previously reported long-distance dispersal of xylem feeders. *Neophilaenus campestris* is able to fly more than 2 km from 35 days during the summer season^31^. Similarly, other spittlebug species, such as *Zulia entrerriana* and *Deois flavopicta,* can travel as much as 3 km^59^. In addition, a back-fitting stochastic model of the epidemiological data in Puglia (southern Italy) revealed that the mode of the long-distance dispersal posterior distribution of spittlebugs is 7 km (Steven White, personal communication). Nevertheless, spittlebugs might be capable of flying with assistance from air currents and travel long distances as they were found flying as much as 200 m above ground^18,22,23^. In addition, hitchhiking is not uncommon and could be another way in which spittlebugs disperse long distances by chance, as has been reported in Italy^39^.

Our approach to estimate the flight activity of *P. spumarius* was by using flight mills under static conditions with no tail wind. In studies based on flight-mill devices, we should consider if the behaviour observed is similar to what we might expect to observe in the field. Tethering could affect insects’ flight behaviour, and the information obtained with flight mills is complex to interpret^35,49,60^. Drag of flight-mill resistive forces, the absence of lifting or something on which to land, flight-related cues and manipulation issues can over- or underestimate the flight capacity of insects being studied^35,57,61,62^. Despite these limitations, flight-mill devices have been used since the 1950s to study the flight performance of a great number of insect species. They are very useful for constructing dispersion models and providing insights that cannot be obtained from more traditional field studies, as we can carry out assays under controlled conditions^32,57,63^.

Approximately 60% of the individuals flew in our seasonal and daily movement assays during the first 15 min since they were attached to the mills. *Philaenus spumarius* has not tarsal reflex and not all individuals start to fly when tethered to flight-mill devices. Thus, they started and stopped flying several times during the recording, so they were not forced to fly until they became exhausted, partially avoiding overestimation of the data^38^.

Our results showed that *P. spumarius* flight activity is influenced by sex and presents daily and seasonal variation. When comparing between sexes, females outperformed males in terms of distance travelled and flight duration. Similarly, the distance travelled, flight duration and mean speed were higher for females than for males during spring. The variation in the flight activity of *P. spumarius* observed in the different seasons seemed to be related to its life cycle. In Mediterranean scrublands, *P. spumarius* adults emerge during spring and feed on herbaceous ground vegetation. As temperature increases during the season, ground vegetation cover dries, forcing insects to continuously disperse and migrate, looking for succulent woody hosts. In autumn, after the first rains, the ground vegetation starts to grow, and spittlebugs leave their oversummering hosts and return for oviposition^44,64^. Our findings about the high flight activity of female *P. spumarius* during spring are consistent with previous findings in which migration behaviour was more active and frequent for sexually immature females than for males^14,17–21^. In some Cicadellidae species, dispersal is greater for sexually immature females than for males^65,66^, and high levels of flight activity in young females was also observed in other spittlebug species^59^.

Our findings show that the flight activity of *P. spumarius* decreased in summer when the insects settle on woody hosts to estivate and increased in early September at the beginning of the oviposition season. A positive correlation between reproductive maturity and migratory flight occurs in some migratory insect species^67,68^. Conversely, a negative correlation or no correlation at all between reproductive maturity and migratory flights exists among some other migratory insects, and even within a same species depending on seasonality^69–71^. According to our results, the increase in the flight activity of both sexes observed in our study during autumn could reflect evolutionary adaptation to increase the probability of encounters between both sexes and maximize mating and fitness. Furthermore, the greater flight activity in females during autumn than during the other seasons could also have been a response to egg maturation and to the need to return to the groves and find suitable host plants for oviposition^18,67^. Considering that *P. spumarius* individuals are able to actively disperse when they become adults and that females exhibit high levels of flight activity during spring, vector control measures should be focused on nymphs and the time before adults start to disperse. In comparison to adults, nymphs are much less mobile and more vulnerable to cultural control actions such as mowing^18^. Although we did not find differences in the flight performance between the populations in Huelva and Madrid (400 km from each other), the influence of climate and landscape should be considered when studying insect movement in the field.

One interesting finding of the present study was related to the daily patterns in the flight activity of *P. spumarius*, with high levels of flight activity during the night. Female flight performance was higher during the night than during the afternoon with no differences in the morning. However, for the males, there were no significant differences in the daily patterns of flight for any of the flight parameters studied. Overall, when compiling male and female data, we found that *P. spumarius* exhibited continuous flight activity throughout the day, but the flight activity was higher during the morning and night than during the afternoon. In fact, we were able to trap *P. spumarius* and *N. campestris* during the night (unpublished data) with yellow sticky traps, but nocturnal dispersal capacity in spittlebugs has never been reported before. Long-distance insect migration is affected by the atmosphere, which can have effects at small scales depending on how wind directions and speeds change during the day^68,72^. The variation in local wind during the day could affect nocturnal insect flight and may lead to unexpected pest outbreaks^73^. Thus, the nocturnal flight activity of *P. spumarius* could impact the daily dispersal process of the vector and should be considered to control the local expansion of *X. fastidiosa*.

The flight performance of *P. spumarius* and the seasonal and daily patterns observed in our study have important implications for control strategies against the spread of *X. fastidiosa* diseases. The boundaries of the buffer zone and the infected zone were recently reduced from 10 km to 2.5–7 km and from 100 to 50 m radius, respectively (EU 2015/789, Articles 4, 5 and 6, 2015; EU 2020/1201, Articles 4 and 5, 2020). Nevertheless, our results revealed that *P. spumarius* is likely to travel much further than the size of the infected and buffer zones established by the EU and that long-distance dispersal is likely to contribute to *X. fastidiosa* spread away from the buffer zones. The eradication of infected and non-infected plants belonging to the same species has a negative impact on many elements in the environment and society^73–74^, and despite this measure, *X. fastidiosa* continues expanding across southern Italy at a rate of 20 km/year^4–6^. Thus, up-rooting uninfected trees may have a limited impact in containing the disease, while identifying (and disrupting) long-distance dispersal processes may be more effective in containing disease epidemics.

In conclusion, considering that *P. spumarius* adults are able to actively disperse during the whole year and that once they acquire the bacterium, they remain infectious over their entire life, one of the critical components of the overall strategy against *X. fastidiosa* should be the management of vector populations in their early stages of development (nymphal stage). This could reduce its spread through long-distance dispersal of its vector.

## Data Availability

The data that support the findings of this study are openly available in: https://doi.org/10.6084/m9.figshare.14229815.
